# Climate Variability Under ENSO Reshapes the *Coffea arabica* Rhizosphere Microbiome While Preserving a Conserved Bacterial Core

**DOI:** 10.3390/plants15081259

**Published:** 2026-04-20

**Authors:** Jorge A. Rueda Foronda, Juan S. Ríos López, Luisa María Múnera Porras, Nancy J. Pino Rodriguez

**Affiliations:** 1 Pollution Diagnosis and Control Research Group (GDCON), School of Microbiology, University of Antioquia, Cl 70 # 52-21, Medellin 050010, Colombia; jorge.rueda1@udea.edu.co (J.A.R.F.); juans.rios@udea.edu.co (J.S.R.L.); 2 Research Group on Health and Sustainability, School of Microbiology, University of Antioquia, Medellin 050010, Colombia; luisam.munera@udea.edu.co

**Keywords:** ENSO, rhizosphere microbiome, *Coffea arabica*, climate variability, soil health, core microbiome

## Abstract

Climate variability is a major driver of belowground microbial assembly, yet its effects on rhizosphere microbiomes in perennial crops remain insufficiently resolved. We investigated how macroclimatic oscillations associated with the El Niño–Southern Oscillation (ENSO) influence bacterial communities in the rhizosphere of *Coffea arabica*. Using 16S rRNA amplicon sequencing across five sampling campaigns covering El Niño, La Niña, and Neutral phases in the Colombian Andes, together with multivariate and variance-partitioning analyses, we quantified the relative contributions of climatic and edaphic factors to rhizosphere community structure. PERMANOVA across three dissimilarity metrics showed that the ENSO explained 11–17% of β-diversity, exceeding the contribution of intra-annual seasonality (6–12%). Ordination analyses indicated moderate compositional differentiation with considerable overlap among ENSO groups, consistent with gradual community turnover under contrasting hydroclimatic conditions. Rainfall and soil pH emerged as the main edaphic correlates of community composition, although their independent effects were no longer significant after accounting for the ENSO phase and season. Despite these shifts, the rhizosphere remained dominated by Acidobacteriota, Actinobacteriota, and Proteobacteria, and a prevalence-defined core microbiome (genera detected in ≥85% of samples) was maintained across climatic phases and seasons. These results indicate that, within the explained fraction of variation, macroclimatic variability contributed more to rhizosphere bacterial turnover than local edaphic heterogeneity, while a conserved prevalence-defined bacterial core may contribute to taxonomic stability in climate-sensitive coffee systems.

## 1. Introduction

The rhizosphere is a dynamic microenvironment where plant roots and soil microorganisms interact, influencing nutrient turnover, soil aggregation, and plant-associated ecological processes [1]. In perennial systems, macroclimatic variability can reshape these interactions, yet few studies have examined their temporal dimension. Seasonal and interannual fluctuations have been shown to reorganize root-associated microbiomes in forest and orchard trees, indicating that community composition is responsive to environmental oscillations rather than solely constrained by host filtering [2,3]. In this study, the ENSO was used as a macroclimatic classification framework to contextualize local hydroclimatic variation, rather than as a direct proxy for all site-level environmental conditions.

*Coffea arabica* sustains the livelihood of over 25 million farmers worldwide and occupies more than 10 million hectares, predominantly in tropical mountain regions highly exposed to El Niño–Southern Oscillation (ENSO) variability [4,5,6]. ENSO-driven droughts and extreme rainfall events strongly affect coffee productivity and soil processes [7], yet their influence on the rhizosphere microbiome remains largely unexplored. Previous studies have described a core bacterial assemblage dominated by Proteobacteria, Acidobacteriota, and Actinobacteriota [8,9], but temporal dynamics across climatic phases have not been quantitatively resolved. While several tropical perennials exhibit seasonally restructured root microbiomes, coffee remains poorly characterized in this regard [10].

Under severe climate change scenarios, only a limited fraction of the land currently suitable for *C. arabica* cultivation, estimated at approximately 15–20%, is projected to remain climatically viable by mid-century, reflecting the species’ high sensitivity to increasing temperatures [7,11,12]. This decline in climatic suitability has been linked to interacting stressors such as drought-induced disruptions of flowering and persistent soil water deficits that constrain root water uptake, resulting in reduced plant performance and yield stability [10,13,14]. Although such impacts highlight the ecological importance of belowground processes, the present study does not directly evaluate plant physiological buffering or microbiome-mediated stress mitigation. Instead, it focuses on whether ENSO-associated hydroclimatic variability is accompanied by detectable restructuring of rhizosphere bacterial community composition.

Here, we tested the hypothesis that ENSO-related hydroclimatic variability is associated with the detectable restructuring of rhizosphere bacterial community composition in *C. arabica*. We further predicted that, despite this turnover, a prevalence-defined recurrent set of taxa would persist across climatic phases and seasons. To address this, we combined 16S rRNA amplicon sequencing with multivariate and variance-partitioning analyses to disentangle the relative influence of macroclimate and soil properties on rhizosphere community structure [15].

## 2. Results

### 2.1. Alpha Diversity

**Figure 1:**
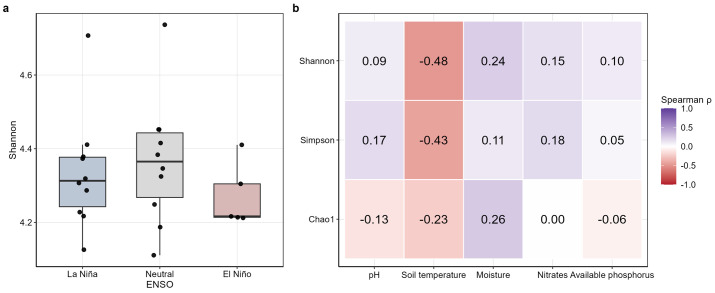
Alpha diversity of bacterial communities in the rhizosphere of *C. arabica*. (**a**) Shannon diversity across ENSO phases (La Niña, Neutral, and El Niño). Boxplots show the median, interquartile range, and individual observations; no significant differences were detected among ENSO phases (Kruskal–Wallis, χ^2^ = 2.29, df = 2, *p* = 0.318). (**b**) Spearman correlation heatmap showing the relationships between alpha-diversity indices (Shannon, Simpson, and Chao1) and selected soil physicochemical variables. Cell values indicate Spearman’s ρ coefficients. No correlations remained significant after false discovery rate (FDR) correction.

Bacterial α-diversity in the *C. arabica* rhizosphere remained stable across ENSO phases (La Niña, Neutral, and El Niño), with no significant differences in Shannon diversity among groups (Kruskal–Wallis, χ^2^ = 2.29, df = 2, *p* = 0.318; Figure 1a). Post hoc Dunn tests likewise detected no significant pairwise differences after Benjamini–Hochberg correction (all adjusted *p* > 0.39). Median Shannon values varied within a relatively narrow range across ENSO phases, indicating that overall bacterial richness and evenness were maintained despite macroclimatic variability.

When α-diversity indices were examined in relation to soil physicochemical variables, only weak to moderate associations were observed (Figure 1b). Shannon and Simpson showed negative correlations with soil temperature (Shannon: ρ = −0.49; Simpson: ρ = −0.43), but none of the tested associations remained significant after false discovery rate correction. Consistent with this pattern, linear modeling suggested a negative but non-significant relationship between Shannon diversity and soil temperature (β = −0.058 ± 0.032, *p* = 0.085, R^2^ = 0.124), whereas pH, moisture, nitrate, and available phosphorus showed similarly weak and non-significant associations. Together, these results indicate that α-diversity was comparatively stable across the climatic and edaphic gradients assessed here, and that the apparent negative relationship with soil temperature should be interpreted as a weak, non-significant trend rather than as a robust predictive pattern.

### 2.2. Beta Diversity and Environmental Structuring

Rhizosphere bacterial β-diversity in *C. arabica* was significantly structured by both the ENSO phase and season across all dissimilarity metrics examined. For Bray–Curtis distances, ENSO explained 13.3% of the variation in community composition (PERMANOVA, R^2^ = 0.133, F = 1.81, *p* = 0.002), whereas season accounted for 9.8% (R^2^ = 0.098, F = 2.68, *p* = 0.001). A comparable pattern was recovered with Aitchison distances, for which the ENSO explained 12.2% of the variation (R^2^ = 0.122, F = 1.58, *p* = 0.001) and season explained 6.8% (R^2^ = 0.068, F = 1.77, *p* = 0.001). Jensen–Shannon dissimilarities yielded the strongest partitioning, with the ENSO explaining 17.0% of the variation (R^2^ = 0.170, F = 2.53, *p* = 0.002) and season explaining 12.2% (R^2^ = 0.122, F = 3.61, *p* = 0.001). Across all three metrics, the ENSO consistently accounted for a larger fraction of compositional variation than season, although the total explained variance remained moderate.

**Figure 2:**
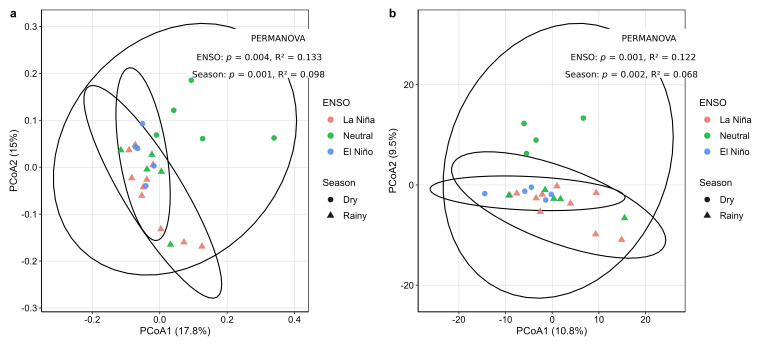
PCoA of rhizosphere bacterial β-diversity in *C. arabica* across ENSO phases and seasonal conditions. (**a**) Bray–Curtis distances showing partial compositional differentiation associated with the ENSO phase and season. PERMANOVA indicated significant effects of the ENSO (R^2^ = 0.133, *p* = 0.002) and season (R^2^ = 0.098, *p* = 0.001). (**b**) Aitchison distances based on CLR-transformed data showing a comparable ordination pattern, with significant effects of the ENSO (R^2^ = 0.122, *p* = 0.001) and season (R^2^ = 0.068, *p* = 0.001). Colors indicate the ENSO phase and symbols indicate season. Ellipses represent 95% confidence intervals. The overlap among groups indicates gradual compositional turnover rather than sharply discrete clustering.

Ordination analyses based on Bray–Curtis and Aitchison distances (Figure 2a,b) showed structured but overlapping distributions among ENSO-defined groups, indicating moderate compositional differentiation rather than sharply discrete clustering. This interpretation was reinforced by the complementary ANOSIM analysis, which yielded low effect sizes and did not detect significant rank-based separation among the ENSO groups for Bray–Curtis (R = 0.024, *p* = 0.317), Jensen–Shannon (R = 0.071, *p* = 0.147), or Aitchison distances (R = 0.103, *p* = 0.071). Together, these results indicate that ENSO-related compositional shifts are detectable but are expressed as gradual turnover across hydroclimatic conditions with substantial overlap among community states.

Consistent with this interpretation, tests of multivariate dispersion did not detect significant heterogeneity among ENSO groups for Aitchison distances (betadisper, *p* = 0.191) or Jensen–Shannon distances (*p* = 0.101), whereas Bray–Curtis showed only a marginal trend (*p* = 0.073). These results suggest that the PERMANOVA patterns primarily reflect differences in community centroid location rather than strong differences in within-group dispersion. When evaluated independently, plot identity also explained a significant fraction of rhizosphere community variation across all three distance metrics, including Bray–Curtis (R^2^ = 0.242, *p* = 0.002), Aitchison (R^2^ = 0.196, *p* = 0.013), and Jensen–Shannon dissimilarities (R^2^ = 0.251, *p* = 0.012), indicating that local spatial heterogeneity also contributed measurably to community structure.

**Figure 3:**
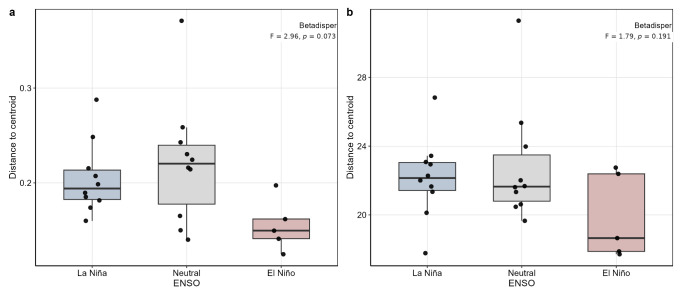
Distances to group centroids across ENSO phases for rhizosphere bacterial communities associated with *C. arabica*. (**a**) Bray–Curtis distances and (**b**) Aitchison distances based on CLR-transformed data. Boxplots show the distribution of within-group distances to centroid for each ENSO phase. Betadisper tests indicated no significant heterogeneity of dispersion for Aitchison distances (F = 1.79, *p* = 0.191), whereas Bray–Curtis showed only a marginal trend (F = 2.96, *p* = 0.073).

Tests of homogeneity of multivariate dispersion across ENSO phases (Figure 3a,b) were non-significant for Aitchison distances (betadisper, *p* = 0.191) and Jensen–Shannon distances (*p* = 0.101), whereas Bray–Curtis showed only a marginal trend (*p* = 0.073). These results indicate that the significant PERMANOVA patterns are unlikely to be driven primarily by heterogeneity in within-group dispersion.

**Figure 4:**
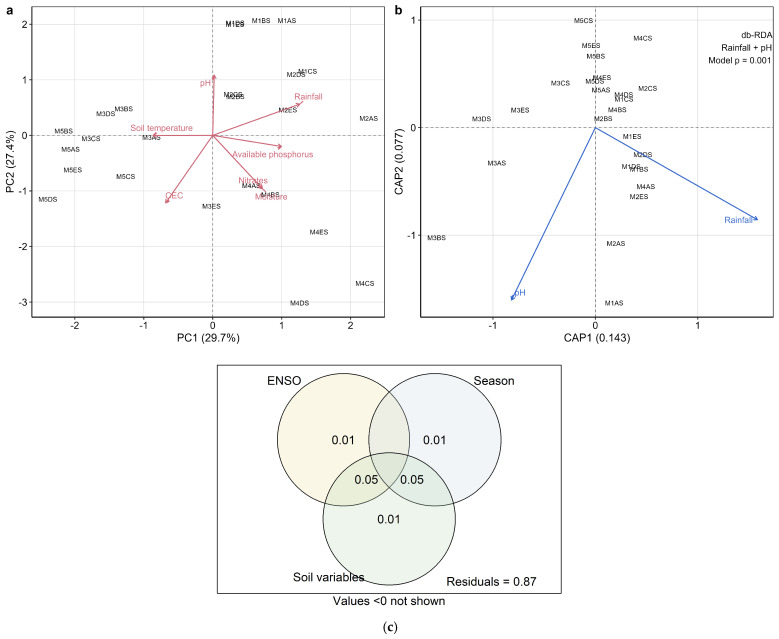
Environmental drivers of rhizosphere β-diversity in *C. arabica*. (**a**) Principal Component Analysis (PCA) of measured soil physicochemical variables across sampling plots and seasons. Arrows represent environmental variables. The first two principal components explain 57.1% of the total environmental variation (PC1 = 29.7%, PC2 = 27.4%). (**b**) Distance-based redundancy analysis (db-RDA) based on Bray–Curtis dissimilarities showing the relationship between rhizosphere bacterial community composition and environmental gradients retained by forward selection. The constrained axes (CAP1 and CAP2) represent the main environmental gradients associated with compositional turnover. (**c**) Variance partitioning of Bray–Curtis distances showing the unique and shared adjusted R^2^ fractions explained by the ENSO phase, season, and soil physicochemical variables. The non-overlapping areas represent the unique contribution of each component, whereas overlapping areas indicate shared explained variation among macroclimatic, seasonal, and edaphic factors. Macroclimatic and seasonal components together account for a larger proportion of the explained variation than soil variables alone, although most variance remains unexplained.

To explore covariance among measured soil physicochemical variables, a principal component analysis (PCA) was first performed (Figure 4a). The first two axes explained 57.1% of the total environmental variation (PC1 = 29.7%, PC2 = 27.4%). PCA was used here as an exploratory dimensionality-reduction tool to visualize the main environmental gradients across samples. The original environmental variables were then used in distance-based redundancy analyses (db-RDA) to preserve direct ecological interpretability of individual predictors in the constrained ordinations.

The global db-RDA model was significant for both Bray–Curtis dissimilarities (F = 1.44, *p* = 0.006) and Aitchison distances (F = 1.26, *p* = 0.001). In marginal tests, soil pH emerged as the strongest environmental predictor in both frameworks (Bray–Curtis: *p* = 0.056; Aitchison: *p* = 0.052), whereas soil temperature showed a weaker, non-significant tendency (Bray–Curtis: *p* = 0.100; Aitchison: *p* = 0.180). Forward selection under the Bray–Curtis framework retained rainfall and pH as significant predictors, together explaining 17.8% of the total inertia, with eigenvalues of 0.143 for CAP1 and 0.077 for CAP2. In the resulting db-RDA ordination (Figure 4b), the main constrained gradients were aligned with rainfall and pH, indicating that these variables captured the most parsimonious environmental structure associated with compositional turnover.

When the db-RDA model was conditioned on the ENSO phase and season, the residual effect of edaphic variables was no longer significant for either Bray–Curtis (*p* = 0.397) or Aitchison distances (*p* = 0.298). Variance partitioning further supported this interpretation (Figure 4c): the unique fractions attributable to the ENSO, season, and soil variables were small (adjusted R^2^ = 0.011, 0.015, and 0.008, respectively), whereas a larger proportion of the explained variation was associated with shared fractions among these components. Overall, these results indicate that rhizosphere bacterial turnover was associated with broader hydroclimatic variability linked to the ENSO and seasonal dynamics, while edaphic gradients contributed mainly through shared environmental effects. Because the unique fractions attributable to the ENSO, season, and soil variables were small and much of the total variation remained unexplained, the climatic signal should be interpreted within the measured explanatory framework rather than as exhaustive of the processes shaping community composition.

### 2.3. Taxonomic Composition of the Rhizosphere Bacterial Community

**Figure 5:**
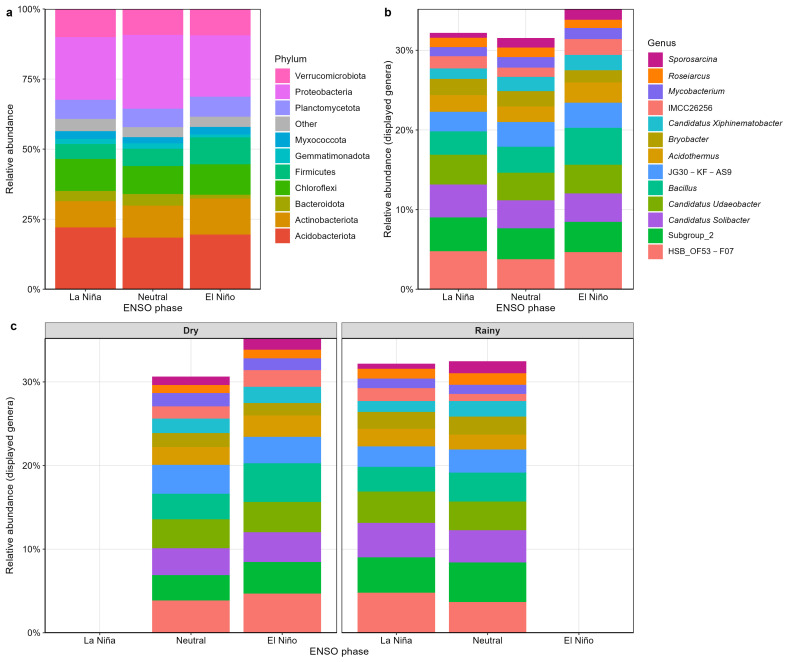
Taxonomic composition of the rhizosphere bacterial community. (**a**) Relative abundance of dominant bacterial phyla across ENSO phases (La Niña, Neutral, and El Niño). (**b**) Relative abundance of the dominant displayed genera across ENSO phases, highlighting the recurrent presence of highly abundant taxa such as HSB_OF53-F07, Subgroup_2, Candidatus Solibacter, Candidatus Udaeobacter, and Bacillus. (**c**) Relative abundance of the dominant displayed genera across ENSO phases stratified by season (Dry and Rainy). Panels (**b**) and (**c**) show the subset of dominant genera selected for visualization.

Across all samples, the *C. arabica* rhizosphere was dominated by members of Proteobacteria, Acidobacteriota, Actinobacteriota, Chloroflexi, and Firmicutes, which together accounted for approximately 80–85% of the total relative abundance (Figure 5a). Minor yet recurrent phyla such as Planctomycetota, Verrucomicrobiota, and Gemmatimonadota were also consistently detected, indicating the presence of bacterial lineages commonly associated with organic-rich tropical soils.

At the genus level, the dominant displayed taxa across ENSO phases were HSB_OF53-F07, Subgroup_2, *Candidatus* Solibacter, *Candidatus* Udaeobacter, *Bacillus*, and JG30-KF-AS9 (Figure 5b). Additional recurrent genera, including *Acidothermus*, *Bryobacter*, *Candidatus xiphinematobacter*, IMCC26256, *Mycobacterium*, *Roseiarcus*, and *Sporosarcina*, were detected at lower relative abundance but remained consistently represented across climatic categories. Together, these profiles indicate that the rhizosphere microbiome was characterized by a stable set of recurrent dominant genera rather than abrupt taxonomic replacement among ENSO phases.

Relative abundance patterns at the phylum level suggested only subtle visual differences among ENSO phases. During El Niño, Firmicutes appeared slightly higher, whereas Acidobacteriota tended to be relatively higher during La Niña (Figure 5a). Proteobacteria remained the most abundant phylum across all climatic phases, with only modest variation in relative abundance. These observations are descriptive and were not evaluated using formal differential abundance testing; they should therefore be interpreted cautiously.

When the dominant displayed genera were stratified by season (Figure 5c), compositional differences were modest and generally smaller than those observed among ENSO phases. The most abundant genera remained broadly consistent across dry and rainy conditions, with only minor changes in their relative contribution. Overall, the *C. arabica* rhizosphere harbors a taxonomically stable bacterial community dominated primarily by Proteobacteria and Acidobacteriota at the phylum level, while a conserved set of dominant genera persists across hydroclimatic phases, supporting the interpretation of taxonomic continuity in the rhizosphere microbiome across hydroclimatic categories.

### 2.4. Core Microbiome of the Coffee Rhizosphere

**Figure 6:**
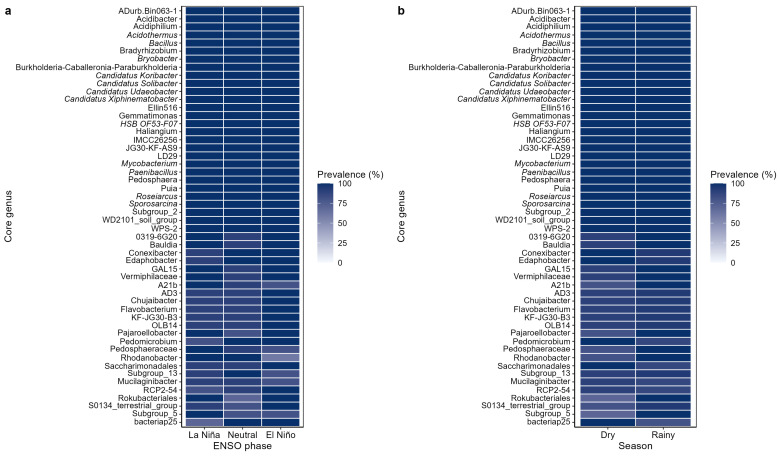
Prevalence patterns of core genera in the *C. arabica* rhizosphere. (**a**) Prevalence (%) of globally recurrent core genera across ENSO phases (La Niña, Neutral, and El Niño). The near-overlapping prevalence values across climatic categories indicate high similarity and minimal turnover of the core microbiome among ENSO phases. (**b**) Prevalence (%) of the same core genera across seasonal categories (Dry and Rainy), likewise showing strong persistence across seasons. Core genera were defined as taxa detected in at least 85% of all samples. Darker colors indicate higher prevalence.

The *C. arabica* rhizosphere hosted a highly prevalent bacterial core comprising 52 genera detected in at least 85% of all samples (Figure 6a,b). Many of these taxa exhibited complete prevalence across the dataset, including ADurb.Bin063-1, *Acidibacter*, *Acidiphilium*, *Acidothermus*, *Bacillus*, *Bradyrhizobium*, *Bryobacter*, *Burkholderia-Caballeronia-Paraburkholderia*, *Candidatus koribacter*, and *Candidatus solibacter*, among others. This broad and recurrent taxonomic set indicates that the coffee rhizosphere contains a prevalence-defined recurrent bacterial backbone shared across samples.

Across ENSO phases, prevalence patterns remained remarkably stable. Most core genera were detected in 90–100% of samples within La Niña, Neutral, and El Niño categories, with only minor fluctuations among phases (Figure 6a). Likewise, seasonal comparisons revealed similarly high persistence, with most core genera maintaining high prevalence in both Dry and Rainy samples (Figure 6b). Although a few taxa showed slightly lower prevalence in one category than in another, the overall pattern was one of strong recurrence rather than phase-specific turnover.

Several recurrent genera identified as abundant in the community composition analyses—such as HSB_OF53-F07, Subgroup_2, *Candidatus solibacter*, *Candidatus udaeobacter*, *Bacillus*, *Acidothermus*, *Bryobacter*, *JG30-KF-AS9*, *Roseiarcus*, and *Sporosarcina*, also displayed consistently high prevalence across hydroclimatic conditions. Together, these results support the existence of a highly conserved rhizosphere core microbiome whose membership remains broadly stable despite macroclimatic variability associated with ENSO and seasonal transitions.

Overall, the prevalence structure of the core microbiome indicates that the *C. arabica* rhizosphere is not organized around narrowly condition-specific taxa, but rather around a persistent and widely shared assemblage of bacterial genera. Because this core was defined exclusively by prevalence and not by abundance, ecological centrality, or functional relevance, it should be interpreted as a prevalence-defined recurrent community fraction rather than as direct evidence of functional stability. Within this framework, the recurrence of these genera across ENSO phases and seasons suggests persistent taxonomic recurrence under fluctuating hydroclimatic conditions.

## 3. Discussion

### 3.1. Climatic Variability as an Important Driver of Rhizosphere β-Diversity

Across all dissimilarity metrics, the ENSO explained a larger proportion of rhizosphere β-diversity than intra-annual seasonality, indicating that macroclimatic variability was a stronger correlate of bacterial community turnover in *C. arabica* soils than short-term seasonal change in this dataset. This pattern suggests that broad hydroclimatic oscillations act as an ecological filter on rhizosphere assembly above the scale of local temporal variation. Such an interpretation is consistent with current conceptual frameworks in plant microbiome ecology, which recognize climate and moisture–temperature regimes as major external drivers of belowground community assembly in perennial systems, where host identity remains relatively stable over time [1]. At the same time, because a substantial proportion of β-diversity remained unexplained, this climatic effect should be interpreted as important within the measured explanatory framework rather than as exhaustive of the processes shaping rhizosphere community composition.

Importantly, the ordination patterns do not support an interpretation based on sharply discrete clustering of ENSO-defined microbiomes. Instead, the PCoA results indicate moderate compositional differentiation with substantial overlap among groups. This interpretation is reinforced by the complementary ANOSIM, which yielded low effect sizes and did not detect significant rank-based separation among ENSO categories despite the significant PERMANOVA results. Taken together, these findings indicate that ENSO-associated restructuring in the *C. arabica* rhizosphere is better understood as a gradual turnover in community composition rather than as the emergence of fully distinct microbial states.

Comparable patterns have been described in longitudinal studies of perennial plant microbiomes, where seasonal and interannual environmental variation generates reproducible yet only moderately differentiated changes in community composition [2,3]. In orchard-grown apple trees, for example, root-associated bacterial communities showed clear spatial and temporal structuring across sampling campaigns, while remaining embedded within a persistent host-associated context [2]. Likewise, studies in *Populus* have shown that microbiome composition tracks both seasonal fluctuation and longer-term temporal development, indicating that temporal turnover in perennial systems is structured and repeatable rather than random [3]. These comparisons are intended as ecological analogies rather than as direct equivalents, given the clear differences between managed coffee agroecosystems and natural or orchard tree systems in terms of host management, soil disturbance, and agronomic context. Within this broader context, the ENSO signal observed here appears ecologically plausible and consistent with a climate-responsive, but not abruptly reconfigured, rhizosphere microbiome [16].

### 3.2. Macroclimatic Variability and Edaphic Context Jointly Structure the Coffee Rhizosphere Microbiome

The environmental analyses refine this interpretation by showing that edaphic effects, although detectable, were strongly interrelated with the macroclimatic context captured by the ENSO phase and season. In the db-RDA, rainfall and soil pH emerged as the most informative environmental correlates of community composition, and both variables are ecologically credible structuring factors for rhizosphere communities. Rainfall influences soil aeration, redox conditions, nutrient diffusion, and root exudation dynamics, whereas pH remains one of the most widely recognized determinants of bacterial distribution in soils. However, the contribution of these variables was reduced when the ENSO phase and season were incorporated into the model, and the unique soil-only fraction explained only a small proportion of the total variance [17,18,19].

This result is important because it does not contradict the well-established role of pH in soil microbial biogeography; rather, it places that role within a hierarchical framework. In this coffee system, pH and related edaphic gradients contributed to community structure, but much of their explanatory signal overlapped with broader climatic drivers. In other words, the bacterial assemblage was not organized primarily by local soil chemistry alone, but by hydroclimatic variability acting directly or indirectly through soil environmental conditions. This interpretation is consistent with the variance partitioning results, in which the largest explanatory component resided in the shared fractions among the ENSO, season, and soil variables. Such a pattern suggests that macroclimate and edaphic factors are not independent filters, but interconnected dimensions of the same ecological gradient.

From a mechanistic perspective, this is plausible in tropical mountain coffee systems exposed to the ENSO. Changes in rainfall regime and associated moisture conditions can alter oxygen availability, substrate accessibility, mineralization processes, and rhizosphere diffusion gradients, thereby modifying the conditions under which bacteria compete, persist, or become relatively more abundant [17,18,19]. The fact that rainfall and pH were retained as informative predictors, yet lost independent significance once climate was controlled for, supports the view that local soil conditions may act mainly as proximal mediators of a broader climatic signal rather than as fully autonomous drivers of rhizosphere assembly.

### 3.3. Community Turnover Occurs Without Major Taxonomic Replacement

Although β-diversity analyses revealed significant compositional restructuring, the taxonomic profiles indicate that this turnover occurred without large-scale replacement of dominant lineages. Across all samples, the rhizosphere remained consistently dominated by Proteobacteria, Acidobacteriota, Actinobacteriota, Chloroflexi, and Firmicutes, with only modest shifts in their relative abundance among ENSO phases. At the genus level, the same recurrent dominant taxa remained prevalent across climatic categories, including HSB_OF53-F07, Subgroup_2, *Candidatus solibacter*, *Candidatus udaeobacter*, *Bacillus*, and JG30-KF-AS9. These patterns indicate that climatic variability did not reorganize the coffee rhizosphere through wholesale taxonomic replacement, but through more subtle changes in the relative contribution of already established lineages.

This distinction is ecologically important. In perennial rhizosphere systems, turnover often reflects reweighting within an existing species pool rather than repeated loss and replacement of dominant taxa. The ENSO-associated signal observed here is therefore compatible with a model in which climate-sensitive peripheral or moderately abundant taxa contribute disproportionately to β-diversity, while the dominant taxonomic framework remains comparatively stable. Under such a scenario, community restructuring is real, but it is expressed through shifts in relative abundance and compositional balance rather than through the collapse of one assemblage and its replacement by another [2,3].

This interpretation also helps reconcile the multivariate and taxonomic results. PERMANOVA and ordination analyses indicate that the ENSO phase matters, yet the dominant phyla and genera remain broadly similar across climatic states. Rather than representing a contradiction, this reflects the fact that community composition can change significantly even when high-level taxonomic structure appears stable. In amplicon-based microbiome datasets, particularly those derived from field systems, ecologically meaningful turnover can occur within a comparatively conserved phylogenetic backbone.

### 3.4. A Prevalence-Defined Core Microbiome Underlies Taxonomic Persistence

The clearest evidence of structural persistence in this system comes from the prevalence-based core microbiome analysis. The *C. arabica* rhizosphere hosted a highly prevalent core of 52 genera detected in at least 85% of all samples, and most of these taxa remained present in 90–100% of samples across ENSO phases and seasons. This persistence indicates that the coffee rhizosphere contains a broad and recurrent bacterial assemblage shared across samples rather than being organized around narrowly condition-specific taxa. The stability of this prevalence-defined core is especially notable given that the dataset spans multiple sampling campaigns and contrasting macroclimatic phases.

Several of the taxa that were both prevalent and relatively abundant, such as *Candidatus solibacter*, *Candidatus udaeobacter*, *Acidothermus*, *Bryobacter*, *Bacillus*, *Roseiarcus*, and *Sporosarcina*, have been reported in previous studies of acidic, organic-rich, or nutrient-limited soils and in plant-associated environments [9]. However, the present study does not directly evaluate their functional traits, and their interpretation here should remain compositional rather than functional. Their persistence across hydroclimatic conditions suggests that the coffee rhizosphere maintains a set of bacterial taxa that are either consistently recruited by the host, well adapted to the edaphic context of the system, or both. This interpretation is also consistent with previous work in coffee rhizospheres showing that a relatively small set of prevalent taxa can remain recurrent across geographically distinct production contexts [9].

Importantly, the existence of a conserved core does not imply that the rhizosphere is static. Rather, it suggests that climatic sensitivity and structural stability are not mutually exclusive properties. In this system, ENSO-related variability appears to reorganize the broader community while leaving a persistent set of recurrent taxa largely intact. Because this core was defined exclusively by prevalence and not by abundance, ecological centrality, or functional relevance, it should be interpreted as a prevalence-defined recurrent community fraction rather than as direct evidence of functional stability. Within this framework, the coexistence of broader compositional turnover and recurrence of a shared core is consistent with perennial crop microbiomes, in which long-lived root systems and repeated host filtering may provide continuity even under fluctuating environmental conditions [2,3,9].

### 3.5. Implications, Limitations, and Interpretation of the Hypothesis

From an applied perspective, these findings support the idea that climate-sensitive coffee production may depend not only on managing soil properties, but also on understanding how large-scale climatic oscillations are associated with belowground microbial community reorganization. The recurrent taxa identified here should not be interpreted as immediate inoculant candidates, but rather as ecologically stable lineages that may warrant future isolation, functional characterization, and validation under controlled and field conditions. Their persistence across ENSO phases makes them potentially useful as hypothesis-generating indicators of microbiome stability in coffee systems exposed to hydroclimatic variability.

At the same time, several limitations should be acknowledged. This study is based on amplicon-derived taxonomic profiles and therefore cannot directly resolve the functional consequences of the observed compositional shifts. In addition, although the sampling design captures temporal variation across ENSO phases and seasons, it represents a single coffee production context and a moderate sample size, which limits broader generalization. The relatively low ANOSIM effect sizes further indicate that ENSO-related differentiation is moderate rather than absolute, reinforcing the need for cautious interpretation of ordination-based visual patterns.

Overall, our results support the original hypothesis that ENSO phases and seasonal transitions restructure the bacterial community composition of the *C. arabica* rhizosphere while preserving elements of community stability. This support is nuanced rather than absolute. Climatic variability consistently explained a larger fraction of β-diversity variation than season alone and was associated with detectable compositional turnover, yet the resulting community states remained partially overlapping rather than sharply discrete. At the same time, the persistence of a highly prevalent core microbiome across ENSO phases and seasons indicates that climatic responsiveness is superimposed on a conserved rhizosphere backbone. Together, these findings support a model of climate-responsive community reorganization operating within a taxonomically stable host-associated microbial framework.

## 4. Materials and Methods

### 4.1. Study Site and Sampling Design

Sampling was conducted on a traditional coffee farm (“Finca San Pablo”) located in Ciudad Bolívar, Antioquia, Colombia (5°47′54″ N, 76°03′02″ W). The farm covers approximately 300 ha, of which 25.7 ha are dedicated to *C. arabica* cultivation at elevations ranging from 1600 to 1800 m a.s.l. The area corresponds to a Very Humid Premontane Forest life zone and is characterized by mean annual temperatures between 16 °C and 24 °C and relative humidity exceeding 80%. The dominant coffee varieties were Castillo (80%) and Caturra (20%).

Five 100 m^2^ plots (A–E) were established along a gentle altitudinal gradient (approximately 60 m difference) within the cultivated area. Rhizosphere soil was sampled in five field campaigns conducted between August 2022 and July 2023, with approximately three-month intervals between campaigns in order to capture temporal variation across contrasting hydroclimatic conditions. The campaigns were carried out in August 2022, November 2022, February 2023, April 2023, and July 2023. This temporal structure was designed to encompass both dry and rainy periods and to align the sampling scheme with the prevailing ENSO background during the study period.

Sampling campaigns were classified according to the El Niño–Southern Oscillation (ENSO) phase using the Oceanic Niño Index (ONI), based on sea surface temperature anomalies in the Niño 3.4 region, as reported by the U.S. National Oceanic and Atmospheric Administration (NOAA). ENSO phases were defined following standard criteria: El Niño when the 3-month running mean of Niño 3.4 anomalies was ≥+0.5 °C, La Niña when anomalies were ≤−0.5 °C, and Neutral conditions when anomalies ranged between −0.5 and +0.5 °C. Each sampling campaign was assigned to an ENSO phase based on the ONI value corresponding to the trimester encompassing the sampling date, ensuring that microbial community patterns were interpreted in relation to the prevailing macroclimatic state rather than to an isolated monthly anomaly. Sampling campaigns were also classified as dry or rainy season based on regional precipitation patterns characteristic of the study area. Campaigns conducted during months with historically lower rainfall were assigned to the dry season, whereas those conducted during periods of sustained higher precipitation were assigned to the rainy season.

Within each plot, five rhizosphere subsamples were collected and pooled to form a single composite sample (*n* = 5 plots per campaign). This pooling strategy was adopted to obtain a representative plot-level rhizosphere profile while reducing small-scale microsite heterogeneity among individual plants. However, pooling also limits the ability to quantify within-plot spatial variability, so the resulting dataset should be interpreted as representing plot-level rather than plant-level or microsite-level community structure. Samples were homogenized in the field, transported on ice to the laboratory, and processed immediately upon arrival. Subsamples were stored at −20 °C for DNA extraction and at 4 °C for physicochemical analyses.

### 4.2. Soil Physicochemical Characterization

Air and soil temperature, relative humidity, and light intensity were measured in situ using a digital thermohygrometer (HTC-2) and soil thermometer (Taylor 5976N). Soil pH and moisture were measured with a 3-in-1 sensor (MGMLP1; Hydrofarm, Shoemakersville, PA, USA). Before each sampling campaign, the pH function of the sensor was checked and calibrated against standard buffer solutions (pH 4.0, 7.0, and 10.0) according to the manufacturer’s instructions, in order to ensure consistency of field measurements across campaigns. Because these measurements were obtained under field conditions, they should be interpreted as campaign-specific plot-level measurements rather than as continuous environmental records.

Total nitrogen Kjeldahl (NTC 5889:2011—Icontec Internacional), nitrates and nitrites (internal colorimetric method; parameter reading with SM 4110B:2017), available phosphorus (internal Bray II method), and cation exchange capacity (NTC 5268:2014) were determined in triplicate. Climatic and soil variables were standardized (z-score) for subsequent multivariate analyses.

### 4.3. DNA Extraction, Amplification, and Sequencing

Total genomic DNA was extracted from 250 mg of rhizosphere soil using the QIAamp^®^ PowerFecal^®^ Pro DNA Kit (Qiagen, Hilden, Germany), following the manufacturer’s protocol. DNA purity and concentration were assessed with a NanoDrop™ One spectrophotometer (Thermo Fisher Scientific, Waltham, MA, USA). Extracted DNA samples were shipped on ice to Macrogen Inc. (Seoul, Republic of Korea) for library preparation and sequencing. The hypervariable V3–V4 region of the 16S rRNA gene was amplified using the primers 341F (5′-CCTACGGGNGGCWGCAG-3′) and 805R (5′-GACTACHVGGGTATCTAATCC-3′) [20]. Paired-end sequencing (2 × 300 bp) was performed on an Illumina MiSeq platform.

### 4.4. Data Processing

Raw sequence reads were processed in QIIME2 v2023.9 [21]. Demultiplexed reads were quality-filtered, trimmed, and denoised with DADA2 [22] to generate amplicon sequence variants (ASVs). Non-bacterial sequences (chloroplast and mitochondrial reads) were removed. Multiple sequence alignment was performed using MAFFT [23], and a phylogenetic tree was inferred with FastTree2 [24]. Taxonomic assignment was conducted using a Naïve Bayes classifier trained on the SILVA v138 database [25].

The resulting ASV table, taxonomy, and metadata were exported and analyzed in R v4.5.1 using the phyloseq and microbiome packages. Samples with fewer than 10,000 reads were excluded to ensure adequate sequencing depth. For α-diversity analyses, ASV counts were rarefied to an even depth of 10,000 reads per sample, retaining all samples included in downstream analyses and ensuring adequate coverage of rhizosphere bacterial diversity, as supported by the inspection of rarefaction curves.

For β-diversity analyses, data transformations were applied according to the requirements of each dissimilarity metric. Bray–Curtis and Jensen–Shannon distances were calculated using relative abundance data. For compositional analyses, Aitchison distances were computed as Euclidean distances on centered log-ratio (CLR)–transformed ASV count data after addition of a pseudocount (0.5). Data integrity was verified by inspecting read count distributions and rarefaction curves.

### 4.5. Statistical Analysis

Statistical analyses were conducted in R v4.5.1 [26] using the phyloseq, vegan, microbiome, and ggpubr packages. Analyses were primarily performed at the amplicon sequence variant (ASV) level, with taxonomic agglomeration to the genus level for community summaries and core microbiome analyses. Because each analytical unit corresponded to one pooled composite sample per plot and campaign, all statistical inferences were made at the level of composite plot-by-campaign samples.

Alpha diversity: Shannon, Simpson, and Chao1 indices were calculated using the estimate_richness() function in phyloseq. Differences among ENSO phases (El Niño, La Niña, Neutral), seasons (dry vs. wet), and plots (A–E) were evaluated using Kruskal–Wallis tests, followed by Dunn’s post hoc comparisons with Benjamini–Hochberg false discovery rate (FDR) correction. Pairwise seasonal differences were assessed using Wilcoxon rank-sum tests. Associations between α-diversity indices and continuous soil variables (pH, temperature, moisture, nitrate, and available phosphorus) were examined using Spearman’s rank correlation and simple linear regression.

Beta diversity: Community dissimilarities were quantified using Bray–Curtis, Jensen–Shannon, and Aitchison distances. Bray–Curtis and Jensen–Shannon dissimilarities were calculated on relative abundance data, whereas Aitchison distances were computed as Euclidean distances on centered log-ratio (CLR)–transformed ASV counts (pseudocount = 0.5), explicitly accounting for the compositional nature of amplicon sequencing data. Ordination patterns were visualized using Principal Coordinates Analysis (PCoA) with 95% confidence ellipses.

Group effects of the ENSO phase and season on community composition were tested using permutational multivariate analysis of variance (PERMANOVA; adonis2, 9999 permutations). Because the same plots were sampled repeatedly across campaigns, sampling campaign was treated as a temporal blocking factor for the permutation structure (strata = campaign). This approach constrained permutations within campaigns and reduced inflation of significance due to temporal non-independence. The ENSO phase and season were included as fixed factors, while plot identity was evaluated separately to assess potential local spatial effects within the single-farm design. Sampling campaign was used to preserve the temporal structure of the dataset during permutation, but it was not modeled as an additional fixed factor in the main PERMANOVA models because campaign-level structure was partially aligned with the ENSO classification used as the macroclimatic framework of interest. As a complementary rank-based assessment of group differentiation, analysis of similarities (ANOSIM) was also performed on Bray–Curtis, Jensen–Shannon, and Aitchison distance matrices using 9999 permutations. ANOSIM R statistics and permutation-based *p*-values were used to evaluate whether between-group dissimilarities exceeded within-group dissimilarities.

Core microbiome: The core microbiome was defined at the genus level based exclusively on prevalence criteria. Genera detected in ≥85% of all samples were considered members of the core community. No minimum relative abundance threshold was applied, allowing the identification of persistent taxa irrespective of their proportional contribution, in line with prevalence-based definitions of core microbiomes. Core composition was subsequently compared across ENSO phases and seasons.

To estimate the contribution of the core microbiome to total community abundance, all genera meeting the ≥85% prevalence threshold were first identified in the full dataset. For each sample, the relative abundances of these core genera were then summed to obtain the proportion of the bacterial community represented by the prevalence-defined core. These sample-level values were subsequently summarized descriptively across the full dataset and across climatic categories in order to evaluate the extent to which recurrent taxa accounted for overall community abundance.

Environmental drivers and variance partitioning: Principal Component Analysis (PCA) was used as an exploratory ordination to summarize covariance among measured environmental variables and visualize their joint structure, whereas db-RDA was performed using the original environmental variables to preserve direct ecological interpretability of individual predictors. Distance-based redundancy analysis (db-RDA) was performed using Bray–Curtis and Aitchison dissimilarities to evaluate the influence of edaphic and climatic variables on β-diversity. Environmental variables were z-standardized and screened for multicollinearity (variance inflation factor, VIF < 5). Significance of global and marginal effects was assessed using permutation tests (9999 permutations). Forward selection (ordiR2step) identified significant predictors, and variance partitioning (varpart) quantified the independent and shared contributions of soil variables, the ENSO phase, and the season.

Sequencing depth and compositionality; Rarefaction to a depth of 10,000 reads per sample was applied exclusively for sequencing coverage assessment and α-diversity estimation. In contrast, β-diversity analyses were conducted using compositional approaches, including CLR transformation for Aitchison distances, without rarefaction, following current best practices for amplicon-based microbiome analyses.

## 5. Conclusions

Climatic variability associated with the El Niño–Southern Oscillation was associated with measurable turnover in the *C. arabica* rhizosphere bacterial community and explained a larger fraction of β-diversity than intra-annual seasonality or individual edaphic predictors in this dataset. However, this effect was expressed as moderate compositional differentiation with substantial overlap among community states rather than as sharply discrete clustering.

Despite this climatic signal, the overall taxonomic structure of the rhizosphere remained broadly consistent. Dominant bacterial phyla were maintained across climatic phases, and a highly prevalent 52-genus core persisted across ENSO categories and seasons. These findings indicate that the coffee rhizosphere combines climatic responsiveness at the level of community composition with persistence of a recurrent taxonomic backbone.

Future studies integrating metagenomic, transcriptomic, cultivation-based, and ecophysiological approaches will be necessary to determine how these compositional patterns relate to functional resilience. Extending similar analyses to additional perennial tropical crops will help assess the broader generality of these patterns beyond the single-site context examined here.

## Data Availability

The original contributions presented in this study are included in the article. Further inquiries can be directed to the corresponding author.
